# Impact of Phosphoproteomics in the Era of Precision Medicine for Prostate Cancer

**DOI:** 10.3389/fonc.2018.00028

**Published:** 2018-02-16

**Authors:** Johnny R. Ramroop, Mark N. Stein, Justin M. Drake

**Affiliations:** ^1^Cancer Metabolism and Growth Program, Rutgers Cancer Institute of New Jersey, New Brunswick, NJ, United States; ^2^Developmental Therapeutics/Phase I Program, Rutgers Cancer Institute of New Jersey, New Brunswick, NJ, United States; ^3^Department of Medicine, Division of Medical Oncology and Rutgers Robert Wood Johnson Medical School, New Brunswick, NJ, United States; ^4^Department of Pharmacology, Rutgers Robert Wood Johnson Medical School, New Brunswick, NJ, United States

**Keywords:** clinical trials, kinases, kinase inhibitors, signaling pathways, phosphoproteomics, prostate cancer, mass spectrometry, targeted mass spectrometry

## Abstract

Prostate cancer is the most common malignancy in men in the United States. While androgen deprivation therapy results in tumor responses initially, there is relapse and progression to metastatic castration-resistant prostate cancer. Currently, all prostate cancer patients receive essentially the same treatment, and there is a need for clinically applicable technologies to provide predictive biomarkers toward personalized therapies. Genomic analyses of tumors are used for clinical applications, but with a paucity of obvious driver mutations in metastatic castration-resistant prostate cancer, other applications, such as phosphoproteomics, may complement this approach. Immunohistochemistry and reverse phase protein arrays are limited by the availability of reliable antibodies and evaluates a preselected number of targets. Mass spectrometry-based phosphoproteomics has been used to profile tumors consisting of thousands of phosphopeptides from individual patients after surgical resection or at autopsy. However, this approach is time consuming, and while a large number of candidate phosphopeptides are obtained for evaluation, limitations are reduced reproducibility, sensitivity, and precision. Targeted mass spectrometry can help eliminate these limitations and is more cost effective and less time consuming making it a practical platform for future clinical testing. In this review, we discuss the use of phosphoproteomics in prostate cancer and other clinical cancer tissues for target identification, hypothesis testing, and possible patient stratification. We highlight the majority of studies that have used phosphoproteomics in prostate cancer tissues and cell lines and propose ways forward to apply this approach in basic and clinical research. Overall, the implementation of phosphoproteomics *via* targeted mass spectrometry has tremendous potential to aid in the development of more rational, personalized therapies that will result in increased survival and quality of life enhancement in patients suffering from metastatic castration-resistant prostate cancer.

## Current Treatment Landscape of Prostate Cancer (PrCa)

**Figure 1:**
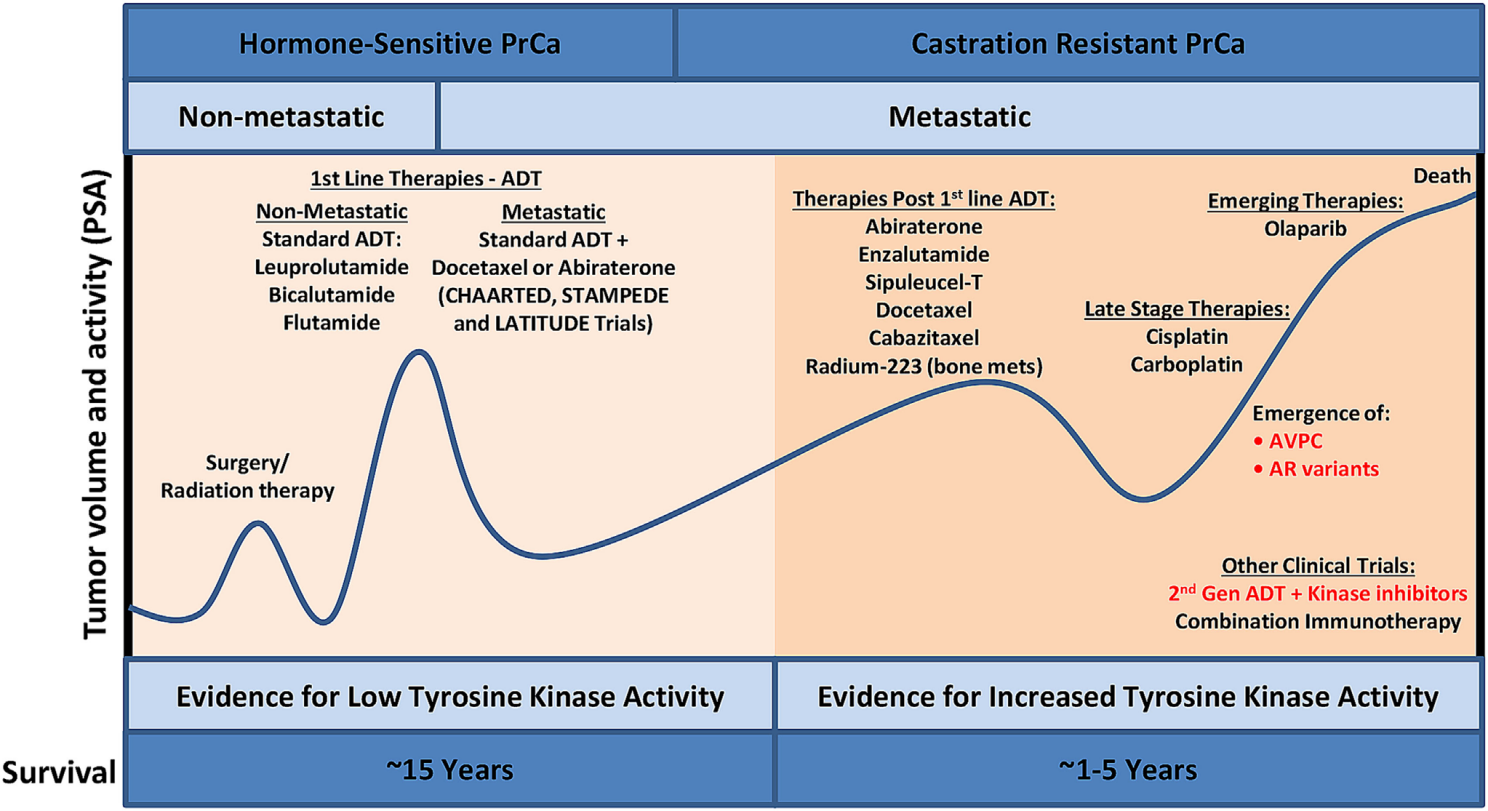
PrCa progression and the current treatment landscape. Despite the availability of effective treatment for PrCa in its early stages, there are constant cycles of regression and recurrence due to therapeutic resistance *via* bypass mechanisms. Utilizing phosphoproteomics approaches to identify activated kinases in late-stage aggressive disease and precisely targeting these kinases with FDA-approved kinase inhibitors, in combination with other standard of care treatment, will lead to increased overall survival. ADT, androgen deprivation therapy; AVPC, aggressive variant prostate cancer; FDA, Food and Drug Administration; PrCa, prostate cancer; PSA, prostate-specific antigen.

The male prostate, as well as early stage PrCa, is dependent on androgens activating the androgen receptor (AR) for survival, growth, and proliferation (1). Prostate-specific antigen (PSA) is a serine protease that is secreted from the prostate and is transcriptionally regulated by AR. Thus, along with digital rectal exams, PSA-based screening is routinely used for early detection of PrCa (Figure 1) although the magnitude of benefit from PSA screening continues to be debated (2). Biopsies are typically done to confirm PrCa, and if diagnosed with clinically significant disease (3), a patient most commonly has external beam radiation therapy or radical prostatectomy. Adjuvant androgen deprivation with radiation or adjuvant radiation after surgery may be administered in the setting of tumors with high risk of recurrence. Androgen deprivation therapy (ADT) that suppresses testicular function is typically first-line therapy for androgen-sensitive PrCa. A small additional survival benefit may be seen when first-generation oral AR inhibitors such as bicalutamide and flutamide are combined with ADT. Initial positive response to ADT is common by evidence of decline PSA levels in 90% of patients (4) and duration of response to therapy varies, with 5–10% of patients surviving 10 years after initiating ADT (5). In two recent phase III clinical trials (STAMPEDE and LATITUDE), hormone naive patients with locally advanced or oligometastatic disease starting first-line long-term ADT treatment were also given abiraterone acetate and prednisone (6, 7). Abiraterone acetate is a potent, second-generation inhibitor that blocks androgen synthesis, significantly decreasing circulating androgen below levels achieved with testicular suppression alone (8). In the STAMPEDE trial, inclusion of abiraterone acetate early in the treatment protocol resulted in a lower risk of death by 37%. The 3-year survival rate was 76% with standard ADT treatment alone and 83% with the standard care in combination with abiraterone acetate as well as a 14-month failure-free survival advantage (7). The results were also verified in a separate LATITUDE trial (6). In another phase III clinical trial (CHAARTED), patients with hormone-sensitive metastatic PrCa were given docetaxel in combination with first-line ADT treatment. The median overall survival with combination treatment was 13.6 months longer than with ADT alone (57.6 vs 44 months), nearly identical to the STAMPEDE and LATITUDE trials. A decrease in PSA to <0.2 ng/ml at 12 months was 27.7% for the combination group compared to 16.8% for the ADT only group, and the median time for development of CRPC was increased (20.2 vs 11.7 months) (9). The results of these exciting clinical trials have prompted earlier use of docetaxel or abiraterone acetate plus standard ADT to delay time of metastatic castration-resistant PrCa (mCRPC) and ultimately death.

Metastatic castration-resistant PrCa is associated with poor prognosis with a mean survival time of 16–18 months (10). The US Food and Drug Administration (FDA)-approved therapies for mCRPC include chemotherapy agents (docetaxel and cabazitaxel), second-generation hormonal therapies (abiraterone acetate and enzalutamide), immunotherapy (sipuleucel-T), and radium-223. AR point mutations and amplifications have led to resistance to first-line ADT treatments, and since AR remains active in mCRPC, these patients respond to abiraterone acetate and enzalutamide, but with a modest increase in overall survival of 3–4 months (11). A major resistance mechanism to abiraterone acetate and enzalutamide involve the presence of AR splice variants, such as ARv7 (12), possibly explaining the modest overall survival benefit of these agents in an unselected population. AR splice variants are truncated forms of wild-type AR where the ligand-binding domain is lost, activation is ligand independent, and these variants are constitutively active (13). It was recently shown that ARv7 mRNA detection in circulating tumor cells (CTCs) correlated with poor outcomes in patients with mCRPC who were treated with abiraterone acetate and enzalutamide (14). It is still unclear if AR splice variants are functionally contributing to treatment resistance, but ARv7 has developed into an important predictive biomarker for mCRPC patients taking either abiraterone acetate or enzalutamide. Another result of resistance to prolonged administration of abiraterone acetate and enzalutamide is the development of a lethal variant of mCRPC termed aggressive variant prostate cancer (AVPC; Figure 1). Indeed, AVPC was classified in 15% of mCRPCs prior to the approval of abiraterone acetate and enzalutamide; however, this population shifted to 31% AVPC post-abiraterone acetate and enzalutamide (15). Several great reviews have been written on this disease variant (16–18), and recent work has classified AVPC into two distinct subtypes: AR-null expressing neuroendocrine (NE) differentiation markers and AR-null lacking markers of NE differentiation (double negative) (15). AVPC is characterized by several clinical and genetic features to include low PSA and AR protein expression, loss of retinoblastoma, *TP53* mutations, overexpression of Aurora kinase A (*AURKA*), and amplification of N-Myc (*MYCN*) (19–21). Survival is typically less than a year with limited treatment options to include platinum-based chemotherapy. Based on the recent literature, it is becoming apparent that AR-independent pathways such as activated MAPK and FGFR kinase pathways are responsible for AVPC development and progression. Indeed, several clinical trials investigating AR-independent pathways are underway in PrCa including agents that target MEK and/or SRC (NCT01990196), AURKA (NCT01799278), PI3K/mammalian target of rapamycin (mTOR) (NCT02407054), and DNAPK (NCT01353625), although not all of these trials focus on AVPC.

## Personalized Therapies in PrCa—Clinical Evidence

The treatment landscape of mCRPC and AVPC involve several “one-size-fits-all” approaches with no real stratification of patients or novel targeted therapies, with a few emerging exceptions. The observation that AR amplification and missense mutations are observed in nearly 70% of mCRPC cases (22) has led to the clinical paradigm where all patients are essentially treated with some form of androgen synthesis or AR function inhibitors. Other genetic alterations have been observed at high frequency (ETS gene rearrangements, *TP53* mutations, and *PTEN* deletions) in mCRPC patients but are not yet predictive for any particular targeted therapy. Recent whole-genome and transcriptome sequencing efforts have identified several genetic aberrations in mCRPC patients at lower frequency to include *TP53, RB1, PIK3CA/B, BRAF/RAF1, BRCA2, BRCA1*, and *ATM* (22, 23). In another study by Barbieri et al., the exomes of over 100 primary prostate adenocarcinomas and normal tissue pairs were sequenced and led to the identification of new recurrent mutations including *MED12, FOXA1*, and *SPOP* (24). It was later shown that recurrent point mutations in *SPOP* in PrCa activates the PI3K/AKT/mTOR and AR signaling pathways providing functional evidence that this mutation may serve as a predictive biomarker to PI3K or AKT inhibitors in combination with antiandrogens (25, 26).

Mutations in DNA repair genes (e.g., *BRCA2*) are observed in approximately 10–15% of mCRPC cases (27). In ovarian cancer, FDA-approved poly ADP-ribose polymerase inhibitors, olaparib and rucaparib, have been used for the treatment of ovarian cancer in patients harboring the *BRCA1, BRCA2*, and other DNA repair gene mutations (28) and were sensitive to platinum-based chemotherapies (29). Indeed, recent clinical trials suggest that targeting these DNA repair mutations with PARP inhibitors may also be beneficial in men with mCRPC. In a phase II TOPARP-A trial, treatment with olaparib in patients who stopped responding to standard ADT treatment and who had aberrations in DNA repair genes (notably *BRCA2* and *ATM*) led to a favorable response rate in 88% of patients (30). Response included ≥50% reduction in PSA and reduction in CTCs to <5 per 7.5 ml of blood. Radiographic progression-free survival was longer in biomarker positive patients (median, 9.8 months) compared to biomarker negative (median, 2.7 months). Overall survival was also extended when compared between these two groups (median, 13.8 vs 7.5 months). Based on the data from this phase II TOPARP-A trial, olaparib received FDA breakthrough therapy designation in January 2016 to treat mCRPC patients with *BRCA1/2* or *ATM* mutations who have received prior abiraterone acetate or enzalutamide therapy. Another phase II study is currently underway evaluating the efficacy of rucaparib in patients with mCRPC that harbor mutations in DNA repair genes (NCT02952534). In addition, in *PTEN*-deficient and *TMPRESS2-ERG* expressing PrCa tumor cells, rucaparib addition was synergistic when combined with radiation and suggested the use of rucaparib to radiosensitize PrCa cells as a useful strategy clinically (31). Similarly, other mutations and bypass mechanisms may be targeted to re-sensitize resistant cells or make them radiosensitive. For example, expression of the transcription factor anti-programmed death ligand 1 (AP-1) is associated with mCRPC and constitutively active AP-1 is dependent on EGFR and PI3K. Interestingly, inhibition of PI3K pathway suppresses AP-1 expression, sensitizing PrCa cells to gamma radiation, suggesting a combination of AKT inhibitors with radiation therapy as a novel strategy for treatment (32). Importantly, not all mCRPC patients with mutations in DNA repair genes will respond to olaparib due to secondary mutations that restore wild-type function or other activated cell survival pathways (33). Whole exome sequencing of circulating cell-free DNA (collected during the TOPARP-A trial) suggested that germline and somatic DNA repair mutations (*BRCA2* and *PALB2*) reverted back in frame as the mechanism behind resistance to olaparib providing a novel platform for assessing predictive biomarkers in this patient cohort (28).

Currently, about 20–30% of mCRPC patients resistant to abiraterone acetate or enzalutamide may benefit from stratification and targeted therapy trials as described above. However, the remaining 70–80% of mCRPC patients are devoid of activating mutations in genes that lead to the “smoking gun” hypothesis identifying obvious targeted therapy applications based on the genomic data alone. As we get better at targeting AR for mCRPC, it is becoming clearer that bypass kinase pathways are important mediators of treatment resistance in mCRPC and AVPC and the development of new tools or utilization of existing ones to identify these pathways are becoming necessary. Indeed, future clinical therapies may rely on the precise targeting of these select kinases in this disease in combination with other agents to prolong survival.

## Tools to Assess Kinase Signaling Pathways in Cancer

### Reverse Phase Protein Arrays (RPPAs) and Immunohistochemistry (IHC)

Antibody-based methodologies for the assessment of signaling networks in CRPC (and other cancers) include both IHC (34, 35) and RPPA (36, 37). These antibody approaches are amenable to use on clinical tissues such as biopsies and do not require specialized instruments. However, these assays can be time consuming with respect to optimizing staining protocols for each antibody (IHC), and only a select few phosphosite-specific antibodies are robust enough and are commercially available, limiting the analysis to a predefined set of targets. Also, neither approach is particularly high throughput as IHC staining typically analyzes one protein at a time and RPPA can measure up to a couple hundred from a given tissue section or biopsy. Overall, RPPAs and IHC-based approaches work very well for detecting known and predefined pathways for a given tumor type and to date are better with low sample amounts. However, they are not well suited for easy customization or discovery-based investigation of a large number of cell signaling pathways such as with mass spectrometry (MS)-based approaches.

### MS-Based Phosphoproteomics

Mass spectrometry is an analytical technique that involves the ionization of a molecule and subsequent detection and identification of the fragmented ions based on its mass-to-charge (m/z) ratio. There are two main MS-based proteomics approaches, top–down and bottom–up. The top–down approach involves analyzing whole proteins, while the bottom–up method involves measuring the peptides from digested proteins. These two approaches can be used for either shotgun or targeted platforms. In both approaches, the sample analytes are injected and eluted from a reverse-phase column, ionized, and sorted based on the m/z ratio within the mass spectrometer instrument. The fragment and parent masses are used to determine the identity of the analyte. While MS instruments are expensive, MS does provide the advantage of high data collection power and has the customizable capability of analyzing small, complex sample amounts with high sensitivity, repeatability, and resolution.

**Figure 2:**
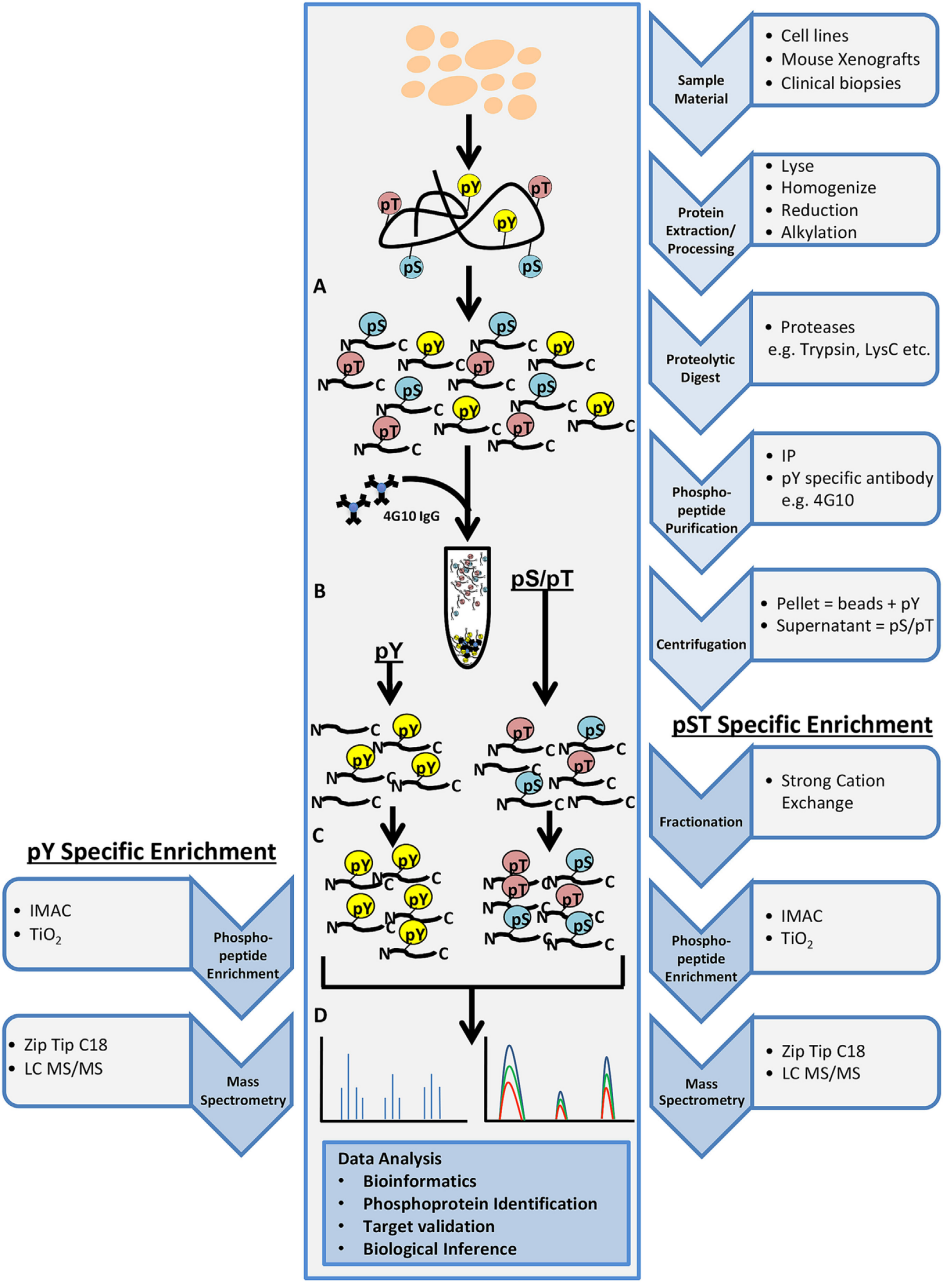
General workflow for shotgun phosphoproteomics analysis. Tissue samples may include cultured cell lines, mouse xenografts, or clinical biopsy specimens. Tissue samples are lysed, homogenized, reduced, alkylated, and digested with the appropriate protease(s) **(A)**. Phosphopeptide purification by immunoprecipitation (IP) and centrifugation will yield two fractions: pellet containing phosphotyrosine peptides (pY) and supernatant containing phosphoserine/phosphothreonine (pS/pT) peptides **(B)**. Strong cation exchange is performed for the pS/pT peptides fraction before phosphopeptide enrichment step for both fractions [immobilized metal affinity chromatography (IMAC) or Titanium oxide (TiO_2_)] **(C)** and analysis by LC-MS/MS **(D)**.

Phosphoproteomics is the identification and characterization of proteins with phosphorylation as a posttranslational modification (PTM). Phosphoproteomics provide insight into proteins that are important for regulating essential signaling pathways and cellular processes and may lead to new potential drug targets. In shotgun phosphoproteomics, protein samples are enzymatically digested into peptides and phosphopeptides (Figure 2A). Digestion is predominantly performed using trypsin because of its high specificity, availability, and ease of use. The limitation of the use of trypsin alone in bottom–up phosphoproteomics is that a comprehensive view of the full phosphoproteome may be compromised as a result of missing particular PTM sites, missed cleavages, or too small peptides. However, LysC can be used in conjunction with trypsin to reduce some missed cleavage events, increasing PTM site coverage. The use of alternative proteases such as chymotrypsin, LysN, AspN, GluC, and ArgC may also help with limitations of trypsin digestion, but these proteases must be used in separate experimental preparations to eliminate the generation of phosphopeptides that are too small for MS detection (38). It was initially believed that up to 30% of all human proteins may be modified by phosphorylation (39). More recent findings indicate that at least approximately 75% of the proteome can be phosphorylated (40). Phosphoserine and phosphothreonine modifications represent approximately 98% of the phosphoproteome (~86 and ~12%, respectively), while tyrosine phosphorylation accounts for approximately 2% of protein phosphorylation in cells (41). Due to the low abundance of phosphopeptides in complex biological tissue, enrichment steps such as immobilized metal affinity chromatography, metal oxide surfaces using titanium oxide (TiO_2_), antibody-based enrichment (e.g., 4G10, used for phosphotyrosine enrichment), or a combination of these approaches are necessary (Figures 2B,C) (42, 43). The digested and enriched phosphopeptides are then analyzed by the mass spectrometer (Figure 2D), and the identified phosphopeptides are collected for data analysis.

While very powerful, shotgun phosphoproteomics does have some limitations. In general, higher abundant phosphopeptides are sampled more frequently, while lower abundant phosphopeptides are sampled less frequently. In addition, high variability of sampling between MS runs can exist as lower abundant phosphopeptides may be sequenced in some samples, but not in others, creating a “missing data” problem that can complicate statistical analyses (44). While thousands of phosphopeptides are identified in complex biological specimens, tool to evaluate and comprehend the large amount of phosphopeptide information is another main challenge. This has led to the development of resources providing a platform for data processing ranging from annotation and pathway enrichment to generating pathway networks and protein–protein interactions such as MaxQuant (45), Skyline (46), and kinase-substrate enrichment analysis (47, 48). A couple excellent reviews describe these software programs in more detail (49, 50) and will not be discussed in this review.

## Phosphoproteomics in PrCa

Mass spectrometry-based proteomics and phosphoproteomics on cell lines, mouse xenografts, and clinical tissue samples have been used to characterize a wide array of different cancer types as well as identify tumor associated signatures that are involved in cancer progression or resistance to standard therapies. Here, we discuss some of the insights gained from MS-based proteomics and phosphoproteomics in PrCa as well as other cancers such as ovarian, lung, breast, and colorectal.

In a previous study by our group, nearly 50% of CRPC tissues showed increased levels of overall tyrosine phosphorylation compared to hormone naive PrCa (51), suggesting that CRPC tissues are prime candidates for investigating the role of activated kinases driving resistance to hormonal therapies. Since there are no clear common mutational drivers explaining this observed increase in tyrosine phosphorylation in CRPC, we discuss some landmark papers that utilized MS-based phosphoproteomics that identified activated kinase pathways, nominating new therapeutic targets in this disease.

Our group previously identified over 8,000 unique phosphopeptides in mCRPC patient tumors obtained at rapid autopsy using MS-based phosphoproteomics (48, 52). The phosphopeptide signatures differentiated treatment naive PrCa from mCRPC and suggested that metastatic sites within the same patient were highly similar in their signaling networks but differed across patients (52). Some of the activated kinases identified include SRC, EGFR, MEK1, JAK2, AKT, MAPK1/3, and PI3K. Further, we connected both SRC and MAPK1/3 activity to nearly 70% of the mCRPC patients who were evaluated in this study, suggesting that a combination of targeted agents to these two kinases may be beneficial clinically if administered to the correct patient population.

Our group also demonstrated that the integration of our phosphoproteomic datasets with genomic and transcriptomic data provided additional pathway information relevant to signaling networks in mCRPC. By using Tied Diffusion through Interacting Events (53) to integrate differentially expressed transcriptional regulators, genomic mutations, and activated kinases in mCRPC, a list of signaling networks with druggable kinase pathways were generated. From MSigDB gene sets, after incorporating phosphoproteomics data, six major signaling pathways were found to be upregulated in mCRPC tumors including the cell signaling pathway, DNA repair pathway, MAPK/AKT/mTOR, and nuclear receptor pathway (48). When the phosphoproteomics data were not included, these signaling pathways were not as highly enriched. Therefore, the integration of phosphoproteomic data enhanced and validated pathway networks determined by genomic and transcriptional analyses. In addition, we developed a patient-specific, rank order list of kinases predicted to drive the mCRPC tumors in each patient. Since we previously showed patterns of intrapatient similarity of kinase signaling, the collection of an easy accessible biopsy may be all that is needed to identify the activated pathways in each patient.

Our group also began to functionally assess these targets in preclinical models of metastatic development. Over 100 kinases were prioritized based on the phosphoproteomic, gene expression, or genomic information in mCRPC tissues and evaluated in a metastatic tail vein model *via* overexpression in non-metastatic mouse cell lines. We found that 20 kinases promoted metastases in an *in vivo* lung colonization screen. In a second metastatic screen, we overexpressed these 20 kinases in human RWPE-1 cells and identified RAF family (ARAF, BRAF, and CRAF), MERTK, and NTRK2 tyrosine kinases to promote bone and visceral metastases (54). These data suggested the potential contribution of wild-type kinases in PrCa metastasis and identified some candidates for future preclinical and clinical targeting.

Since our initial publications assessing the phosphoproteome of both mouse and human tumors, other groups have begun using phosphoproteomics to address different aspects of PrCa biology. By using quantitative MS-based phosphoproteomics of PrCa cell lines DU145 and PC3, an increase in activated focal adhesion kinase (FAK) at residues Y397 and Y596 was observed in docetaxel resistant DU145 (FAK Y397) and PC3 (FAK Y596) cell lines (55). The Y397 phosphosite serves as a binding site for proteins such as SRC, SHC, and the regulatory subunit of PI3K, while the Y596 site falls within the central kinase domain. Treatment with the FAK inhibitor PF-00562271 reduced phosphorylation of FAK but not AKT and had no effect on cell viability. Docetaxel alone reduced phosphorylation of FAK and AKT, and when added in combination with PF-00562271, there was an additive effect and a rescue of chemoresistance. These data suggested that in mCRPC patients who became resistant to docetaxel treatment due to increased FAK activation, combination therapy with the FAK inhibitor PF-00562271 and docetaxel may be administered to overcome chemoresistance (55).

In another study, protein phosphorylation in LNCaP PrCa cell line xenografts grown in intact and castrated mice identified hyperphosphorylation of signaling proteins including MEK, LYN, PRAS40, YAP1, and PAK2 (56). Also, analysis of CRPC clinical samples showed increased PAK2 and YAP1 levels. In androgen-independent PC3 xenografts, the PAK2 inhibitor PF-3758309 and YAP1 inhibitor verteporfin inhibited tumor growth (56). These data suggested that PAK2 and YAP1 as possible key players during the transition from a hormone naive to a castration resistance state.

Another study of parental LNCaP cells and an androgen-independent version of LNCaP revealed over 60 phosphopeptides that are involved in androgen-independent PrCa cell growth, including thyroid hormone receptor-associated protein 3 (THRAP3) (57). THRAP3 is a transcription coactivator of AR, and hypophosphorylation of residues S248 and S253 were found in androgen-independent LNCaP cells. In addition, the interacting protein partners in both phosphorylated and unphosphorylated states of THRAP3 were different. By using non-phosphorylatable mutants (S248A/S253A) and phosphomimetic forms (S248D/S253D) of THRAP3, the interacting partners were related to RNA splicing/processing, suggesting that phosphorylated THRAP3 at S248 and S253 regulates transcriptional programs leading to androgen-independent PrCa cell growth.

Other studies using the parental LNCaP PrCa cell line identified phosphopeptides corresponding to several actionable kinase targets. These included BRAF, PIK3C2G, STK39, CDK1, MAPK2, AKT1, PRKD1, casein kinase 1 and 2 (CK2A1, CK2A2), and glycogen synthase kinase 3 (GSK3B) (58–60).

**Figure 3:**
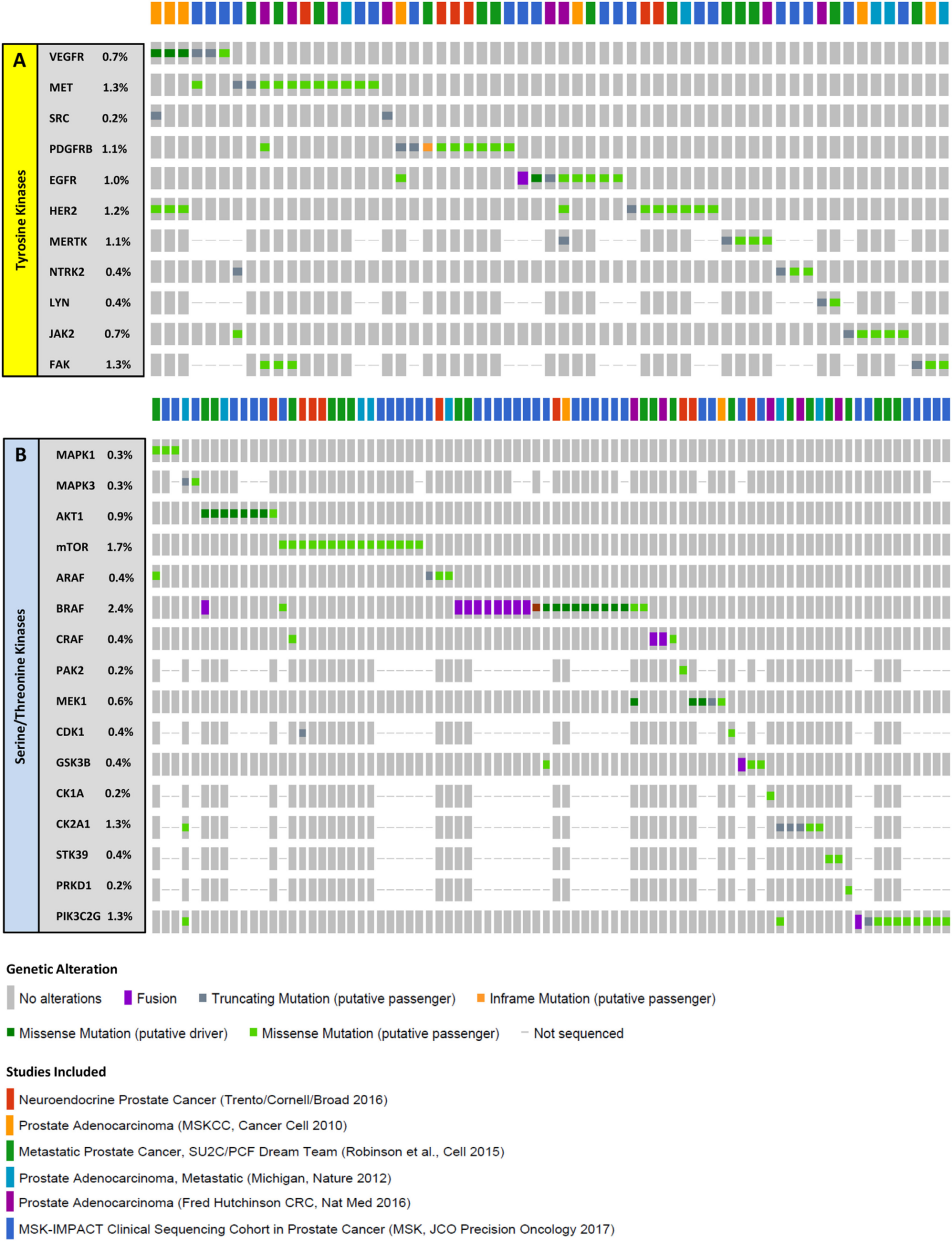
Mutations in select kinases in metastatic castration-resistant prostate cancer (mCRPC). Columns represent individual patients, and rows represent genetic alterations detected in tyrosine **(A)** or serine/threonine **(B)** kinases. For the 6 studies mentioned (22, 23, 67–70), samples from a total of 900 patients were sequenced revealing mutations in 59 patients (~7%) for tyrosine kinases and in 82 patients (~9%) for serine/threonine kinases. Importantly, driver mutations were only observed in 4 patients (~0.4%) for tyrosine kinases and 19 patients (~2%) for serine/threonine kinases, suggesting that a very small fraction of the mCRPC population have genomic identifiers of kinase activity. The proportion of patients with alterations in each kinase is listed on the left. Only patients with alterations are represented. Data were extracted from cBioPortal (71, 72).

The above are most of the examples of work that have demonstrated the value of utilizing phosphoproteomic profiling to reveal regulatory mechanisms and pathways crucial for drug resistance and recurrence in PrCa cell lines and tissues (Table 1). Interestingly, most of these kinases are not mutated at high frequencies at all in mCRPC (Figure 3). In addition to MS, antibody-based proteomic and phosphoproteomics analyses such as IHC (51, 56, 61–63) and RPPA (64–66) have also revealed activated kinases that are involved in disease progression and drug resistance. These identified regulatory proteins and pathways can also serve as potential therapeutic targets.

**Table 1:** Summary of phosphoproteomic studies in prostate cancer.

Kinases and regulatory proteins altered in prostate cancer tissues and cell lines			
Regulatory protein/kinase	Kinase type/function	Source of material	Reference
SRC	Tyrosine kinase	mCRPC patient tumors at rapid autopsy vs treatment naïve primary prostate tissue	Drake et al. (52)
EGFR	Tyrosine kinase	Prostate cancer cell lines: 22Rv1, LNCaP, DU145 and C4-2	Drake et al. (48)
MEK1	Serine/threonine kinase	Prostate cancer cell line derived xenografts: 22Rv1 and LNCaP	
JAK2	Tyrosine kinase		
AKT1	Serine/threonine kinase		
MAPK1	Serine/threonine kinase		
MAPK3	Serine/threonine kinase		
FAK	Tyrosine kinase	Docetaxel resistant DU145 and PC3 prostate cell lines vs Parental DU145 and PC3 prostate cancer cell lines	Lee et al. (55)
MEK1	Serine/threonine kinase	Parental LNCaP prostate cancer cell line	Jiang et al. (56)
LYN	Tyrosine kinase		
YAP1	Transcriptional coactivator		
PAK2	Serine/threonine kinase		
THRAP3	Transcription coactivator	Parental and androgen-independent LNCaP prostate cell lines	Ino et al. (57)
AKT1	Serine/threonine kinase	Parental LNCaP prostate cancer cell line	Giorgianni et al. (58)
BRAF	Serine/threonine kinase		
CDK1	Serine/threonine kinase		
STK39	Serine/threonine kinase		
PIK3C2G	Serine/threonine kinase		
PRKD1	Serine/threonine kinase		
CK1A	Serine/threonine kinase	Parental LNCaP prostate cancer cell line	Myung and Sadar (60)
CK2A1	Serine/threonine kinase		
GSK3B	Serine/threonine kinase		
AKT1	Serine/threonine kinase	Parental LNCaP prostate cancer cell line	Chen et al. (59)
MAPK1	Serine/threonine kinase		
MAPK3	Serine/threonine kinase		

*Some of the potentially key druggable targets identified via MS-based phosphoproteomics that were highlighted in this review for prostate cancer emphasizing the paucity of global phosphoproteomic studies in clinical specimens*.

## Clinical Trials: Kinase Inhibitors and PrCa

Kinase pathways validated from various studies mentioned above would strongly act as clinical biomarkers for the evaluation of patients’ tumor kinase activation profiles. To date, biopsy-driven, therapeutic efforts aimed at targeting wild-type kinases that are activated in mCRPC are not performed. Mounting evidence that cancer is a disease of deregulated signaling pathways has led to the development of signaling-based targeted therapies for various human tumor types based on genomic mutations of these pathways. There are a number of FDA-approved kinase inhibitors for the treatment of cancer such as non-small-cell lung cancer, myeloma, head and neck cancer, and breast cancer, just to name a few (Table 2). These agents target kinases that have also been shown to be activated or upregulated in mCRPC making these inhibitors potentially beneficial to patients with mCRPC if used in the right context or combination. Currently, there are over 900 clinical trials in progress in PrCa in the United States with 85 studies in phase III. Of these, only 21 are investigating kinase inhibitors, alone or in combination, for PrCa. Below are some examples of previous clinical trials utilizing kinase inhibitors and the outcomes.

**Table 2:** List of FDA-approved kinase inhibitors to date with the disease and kinase targets.

Kinase inhibitor	Disease	Kinase target/s
Acalabrutinib	Mantle cell lymphoma	BTK
Afatinib	NSCLC, squamous NSCLC	EGFR, ERBB2, ERBB4
Alectinib	ALK-positive NSCLC	ALK, RET
Axitinib	Renal cell carcinoma	VEGFR1, VEGFR2, VEGFR3, PDGFRβ
Bosutinib	CML	BCR-ABL, SRC, LYN, HCK
Brigatinib	ALK-positive NSCLC after crizotinib	ALK, ROS1, IGF-1R, FLT3, EGFR
Cabozantinib	Metastatic medullary thryoid carcinoma, RCC	RET, MET, VEGFR1, VEGFR2, VEGFR3, KIT, NTRK2, FLT3, AXL, TEK
Ceritinib	ALK-positive NSCLC after crizotinib	ALK, IGF-1R
Cobimetinib	Melanoma with *BRAF* V600E/K mutation with vemurafenib	MEK1/2
Crizotinib	ALK-positive NSCLC, ROS1-positive NSCLS	ALK, MET, ROS1, MST1R
Dabrafenib	Melanoma and NSCLC with *BRAF* V600E	BRAF
Dasatinib	CML, ALL	BCR-ABL, SRC, LCK, YES, FYN, KIT, EPHA2, PDGFRB
Erlotinib	NSCLC, pancreatic cancer	EGFR
Everolimus	ERBB2-negative breast cancer, PNET, RCC, RAML, SEGA	mTOR, FKBP12
Gefitinib	NSCLC	EGFR
Ibrutinib	MCL, CLL	BTK
Imatinib	CML	BCR-ABL, KIT, PDGFR
Lapatinib	Metastatic breast cancer	EGFR, ERBB2
Lenvatinib	Differentiated thyroid cancer	VEGFRs, FGFRs, PDGFR, KIT, RET
Midostaurin	AML with FLT3-positive mutation	FLT3
Neratinib	ERBB2-positive breast cancer	ERBB2
Nilotinib	CML	BCR-ABL, PDGFR, KIT, CSF1R, DDR1
Nintedanib	Idiopathic pulmonary fibrosis	FGFRs, PRGFRα/β, VEGFRs, FLT3
Osimertinib	NSCLC	EGFR T970M
Palbociclib	ER-positive/Her2-negative breast cancer	CDK4/6
Pazopanib	Renal cell carcinoma	VEGFRs, PDGFRα/β, FGFR1/3, KIT, ITK, LCK, FMS
Ponatinib	CML	BCR-ABL, VEGFR, PDGFR, FGFR, EPHR, SRC, KIT, RET, TEK, FLT3
Regorafenib	Colorectal cancer	VEGFRs, BCR-ABL, RET, KIT, FGFR1/2, PDGFRα/β, EPHA2, BRAF
Ribociclib	HR-positive/EGFR-negative breast cancer	CDK4/6
Ruxolitinib	Myelofibrosis, PV	JAK1, JAK2
Sirolimus	Renal transplant lymphangioleiomyomatosis	FKBP12, mTOR
Sorafenib	Hepatocellular, renal, thyroid carcinoma	BRAF, CRAF, KIT, FLT3, RET, VEGFRs, PDGFRβ
Sunitinib	GIST, renal cell carcinoma, PNET	PDGFRα/β, VEGFR1, VEGFRs, KIT, FLT3, CSF1R, RET
Temsirolimus	Advanced renal cell carcinoma	mTOR
Tofacitinib	Rheumatoid arthritis	JAK1, JAK2
Trametinib	Melanoma and NSCLC with *BRAF* V600E mutation	MEK1/2
Vandentinib	Medullary thryoid carcinoma	EGFRs, RET, VEGFRs, TEK, EPHRs, SRC, BRK
Vemurafenib	Melanoma with *BRAF* V600E mutation, Erdheim–Chester disease	BRAF, ARAF, CRAF, SRMS, ACK1, MAP4K5, FGR

*A current (December 2017) list of kinase inhibitors approved for the treatment of various cancer types. Some of these targets are implicated in mCRPC; however, the kinase inhibitors assessed in clinical trials for mCRPC did not demonstrate sufficient response and were not approved*.

*ALL, acute lymphoblastic leukemia; CML, chronic myelogenous leukemia; CLL, chronic lymphocytic leukemia; GIST, gastrointestinal stroma tumor; MCL, mantle cell lymphoma; mCRPC, metastatic castration-resistant prostate cancer; mTOR, mammalian target of rapamycin; NSCLC, non-small-cell lung cancer; PNET, progressive neuroendocrine tumor of pancreatic origin; PV, polycythemia vera; RCC, renal cell carcinoma*.

Bevacizumab, which blocks VEGF signaling, was assessed in a phase III trial comparing the treatment of docetaxel/prednisone alone or in combination with bevacizumab in patients with mCRPC. Combination treatment resulted in a 2.4-month improvement in progression-free survival, but no difference in median survival (73). Sorafenib was shown to prevent disease progression and cause regression of bone metastases in some patients but without decreasing PSA levels (74), and sunitinib also induced a partial radiographic response but had a minimal effect on PSA levels in both treatment naive and docetaxel-treated CRPC patients (75).

Mammalian target of rapamycin inhibitors as a single agent had minimal effect in mCRPC (76), but in combination with docetaxel, it was shown to reverse drug resistance in PrCa cell lines (77). In patients with *PTEN* and other genetic aberrations where the AKT pathway is activated, targeting the AKT pathway in combination with mTOR inhibitors was shown to induce apoptosis. The AKT inhibitor, perifosine, in a phase II trial in patients with recurrent, hormone-sensitive PrCa did not meet prespecified PSA criteria for response with only 20% of patients showing a reduction in PSA, but none had a decline greater than 50%. As a single agent, there was only modest clinical activity with perifosine but other AKT inhibitors are in clinical trials in combination studies with docetaxel (NCT02121639), bicalutamide (NCT01251861), and abiraterone (NCT01485861). Everolimus, another mTOR inhibitor, was FDA approved in 2016 for the treatment of patients with NE tumors of pancreatic origin. In a phase I study, everolimus in combination with docetaxel was found to be safe in CRPC patients and resulted in more than 50% reduction in PSA levels (78). Another investigational mTOR inhibitor, ridaforolimus, in phase II trials in combination with bicalutamide showed a 30% decrease in PSA response (79).

SRC non-receptor tyrosine kinase is involved in multiple signaling pathways in PrCa including cell proliferation, migration, angiogenesis, survival, and transition to androgen-independent growth (80). Dasatinib is a SRC inhibitor that was shown to suppress cell proliferation of PrCa cell lines (81); inhibit cell adhesion, migration, and invasion (82); and reduce tumor growth in a mouse xenograft model (83). In a phase II clinical trial, dasatinib had a definite but limited activity in advanced mCRPC but was poorly tolerated, and 43% of patients discontinued treatment due to toxicity (84). In a previous phase II clinical trial, dasatinib did not show significant PSA response, and only 19% of men with mCRPC were free of disease progression at 6 months (85). In a separate phase II combination trial of dasatinib and docetaxel, it was observed that 41% of the participants showed a PSA response and 32% of patients with bone metastases showed improvement as assessed by bone scans (86). In a phase II clinical trial with single-agent saracatinib, another SRC inhibitor, only 18% of patients with mCRPC showed a reduction of <30% in PSA (87). In a more recent phase II clinical trial, saracatinib was assessed in a subset of mCRPC patients who showed recurrence postdocetaxel treatment. Only 26% of patients had stable disease after 8 weeks with the remaining patients showing expansion of existing lesions. Fatigue was reported in 25% of patients, and this study was discontinued as it could not be determined if saracatinib would inhibit metastasis (88). Another phase II study of a SRC inhibitor, KX2-391, was closed due to prespecified futility rule and toxicities (89).

A plethora of kinase inhibitors have been assessed in phase I and phase II trials for patients with mCRPC. These include single agents such as sorafenib (74), sunitinib (90), cabozantinib (91), dasatinib (85), lapatinib (92), imatinib (93), and gefitinib (94) as well as erlotinib (95) and dasatinib (96) in combination with docetaxel. None of these agents demonstrated sufficient response or activity to advance to phase III trials, with the exception of cabozantinib and dasatinib (Table 3). Unfortunately, neither of these two inhibitors demonstrated significant overall improved survival benefits in those phase III trials (85, 91).

**Table 3:** Kinase inhibitors that have been assessed in clinical trials for mCRPC.

Kinase inhibitors	Target	Approved?	Phase reached	Reference
Cabozantinib	VEGFR, MET	No	III	Smith et al. (91)
Cediranib	VEGFR	No	II	Dahut et al. (97)
Dasatinib	SRC	No	III	Araujo et al. (96)
Dasatinib	SRC	No	II	Yu et al. (85)
Erlotinib	EGFR	No	II	Gross et al. (95)
Gefitinib	EGFR	No	II	Canil et al. (94)
Imatinib	ABL	No	II	Lin et al. (93)
Lapatinib	EGFR, HER2	No	II	Whang et al. (92)
Saracatinib	SRC	No	II	Lara et al. (87)
Saracatinib	FYN	No	II	Posadas et al. (88)
Sorafenib	PDGFR, VEGFR	No	II	Aragon-Ching et al. (74)
Sunitinib	PDGFR, VEGFR	No	II	Dror Michaelson et al. (90)

*Food and Drug Administration approved kinase inhibitors assessed in early phase I and II clinical trials for metastatic castration-resistant prostate cancer did not demonstrate sufficient response or activity to advance to phase III trials, with the exception of cabozantinib and dasatinib. However, neither inhibitor demonstrated significant overall survival benefits and both were not approved*.

Most of the kinase inhibitors that entered clinical trials for mCRPC have been discontinued due to low efficacy even though they have been approved for treatment in other cancer types. This emphasizes the complexity and lack of any one dominant kinase driving the biology in mCRPC, affecting the response of any one targeted therapy in an unselected patient population. The challenge is to develop clinical trials based on biomarkers that can categorize a patient’s cancer subtype to any one of several FDA-approved targeted therapies, similar to the NCI-MATCH trials, but for protein activity. To date, only eight PrCa patients have been “matched” to any given targeted therapy in the NCI-MATCH trial (NCI Formulary, May 8, 2017). This reveals a couple concerns about implementing targeted therapies in mCRPC: (1) the paucity of activating mutations as assessed by genomic panels eliminates a majority of CRPC patients from selected targeted therapies and (2) even if the mutation is observed, information on the activity of that protein is missing. To that point, selection of a targeted agent by mutation information alone, independent of tissue type may also be misleading. Key evidence from colon cancer suggests that BRAF (V600E)-mutant tumors are not responsive to vemurafenib alone but only in combination with an EGFR inhibitor to repress the rapid feedback activation of EGFR by vemurafenib treatment (98). This example suggests that to observe prolonged, clinically significant benefits to patients with mCRPC (or other cancer types), we need to begin designing trials that not only assess the genomic aspects of the tumor but also the feedback regulation. If we can do this, we might have a better chance of inhibiting resistance and prolonging survival.

While disappointing, these clinical trial results do not mean that agents used in these failed clinical trials will not have a future role in mCRPC treatment. It is postulated that select kinase inhibitors used in combination with other targeted agents such as second-generation hormonal therapies (abiraterone acetate and enzalutamide) or checkpoint inhibitor immunotherapies may provide substantial clinical benefit. We are only starting to understand the mechanism of action of these kinase inhibitors in the tumor microenvironment and on immune cells. As this becomes clearer, we can move forward with more rationale combination treatments.

## Proteomics and Phosphoproteomics in Other Cancers

### Ovarian Cancer

In an effort to characterize the genome and proteome of high-grade serous ovarian cancer, Zhang et al. performed MS-based proteomic analysis on 174 ovarian tumors (169 high grade) previously characterized by The Cancer Genome Atlas (TCGA) (99). The integration of genomic and proteomic data showed an 80% positive correlation between mRNA and protein abundance with metabolic pathways and interferon response being more highly correlated than stable, abundant proteins consisting of housekeeping genes. Unbiased clustering using protein abundance data grouped tumors into mesenchymal, proliferative, immunoreactive, and differentiated subtypes, as previously defined by the TCGA transcriptome analysis. However, there was a fifth cluster of tumor-enriched proteins related to extracellular matrix interactions and erythroid and platelet functions that were not identified by genomic data alone. Interestingly, increased protein phosphorylation in high-grade serous ovarian cancer samples suggested that multiple pathways may be activated in these tumors. Indeed, the PDGFR signaling pathway important for angiogenesis, the RHOA regulatory and integrin-linked kinase pathway associated with invasion and cell mobility, and pathways associated with chemokine signaling and adaptive immune response were observed to be activated explaining the aggressive nature of this disease. In patients with activated PDGFR, for example, stratification and enrollment of patients with expected short overall survival into antiangiogenic clinical trial therapies will be beneficial. A phase II clinical trial to determine the effectiveness of imatinib, a PDGFR and KIT inhibitor, in patients with refractory ovarian cancer has been completed (NCT00039585) with no results posted to date. However, it might be promising as imatinib has been shown to be effective to inhibit the growth of ovarian cancer cells in a PDGFR-specific manner by arresting cells at the G0–G1 phase and preventing progression through the S phase (100). Other multifunctional kinase inhibitors that may be effective therapeutic agents for ovarian cancer include pazopanib (targets VEGFR, PDGFR, FGFR, and KIT), sunitinib (targets VEGFR, PDGFR, FLT3, and KIT), and sorafenib (targets VEGFR, PDGFR, and RAF kinases) (101). However, these agents did not do well in phase I and II clinical trials. With pazopanib, there was a 5.6-month progression-free survival and overall survival could not be determined because of toxicities and adverse effects (102). With sunitinib, an objective response rate of only 8.3% with a 9.9-week progression-free survival reported (103). Sorafenib showed only 3.4% partial response, no progression-free survival, or overall survival advantage and low tolerability (104).

### Lung Cancer

Previous work by Rikova et al. have analyzed 41 non-small-cell lung cancer cell lines and more than 150 tumors to identify and group samples based on the activated tyrosine kinases using phosphoproteomics (105). Robust phosphorylation was observed in known oncogenic kinases such as EGFR and c-MET, as well as novel (at the time) ALK and ROS fusion proteins (105). In addition, they identified activated tyrosine kinases not previously indicated in this disease such as PDGFRα and DDR1. PDGFRα, which was found to be aberrantly activated in the H1703 cell line and also in eight tumor samples, was nominated as a novel therapeutic target. Investigating the sensitivity of H1703 cell line to imatinib (PDGFR inhibitor) and gefitinib (EGFR inhibitor) showed that phosphorylation of AKT at serine 473 was blocked by imatinib but not gefitinib. Cell lines negative for PDGFRα were insensitive to imatinib, correlating kinase activity to drug sensitivity. A phase II clinical trial assessed the effectiveness of imatinib and docetaxel in patients with PDFGR expressing non-small-cell lung cancer but was terminated early due to high drug intolerance and no added clinical efficacy. The authors recommend that future studies with PDGFR inhibitors should include the measurement of PDGFR as a positive predictive biomarker prior to therapy administration (106).

Quantitative phosphoproteomics was performed on non-small-cell lung cancer tumors derived from multiple patients, and signaling networks that were known to be involved in lung cancer oncogenesis were identified (107). Activated kinases found in these tumor samples included ERBB2, c-Met, AKT, RAF, and PI3K and direct transcriptional regulators (MEF2D, TP53, ELK3, and ATF7).

### Breast Cancer

Mertins et al. utilized quantitative MS-based proteomics and phosphoproteomics to analyze over 100 genomically characterized breast cancers (108). The samples represented four main mRNA-defined PAM50 breast cancer subtypes, namely basal-like, luminal A, luminal B, and *ERBB2*-positive subtypes. Their results revealed a connection between loss of *CETN3* and *SKP1* to increased expression levels of EGFR and *SKP1* loss to increased SRC levels (108). They also identified a G-protein couple receptor cluster that was not identified at the mRNA level. In addition, highly phosphorylated kinases identified included ERBB2, CDK12, PAK41, RIPK2, and TLK2. The proteome subtypes identified by using the global proteomics and phosphoproteomics data included basal-enriched, luminal-enriched, and stromal-enriched, while *ERBB2*-enriched tumors were distributed among these three proteome subgroups (unlike with the clustering seen with the PAM50 genes, mRNA-defined subtypes). The basal and luminal groups showed a significant overlap between mRNA and proteome-defined subtypes, but the stromal-enriched proteome subgroup represented a mix of all mRNA-based subtypes. Pathway analyses showed that the luminal subtype was exclusively enriched for estradiol and ESR1-driven genes, while the basal proteome subtype was enriched for multiple gene sets including MYC target genes for cell cycle, checkpoint, and DNA repair pathway regulators including AURKA/B, ATM, ATR, CHEK1/2, and BRCA1/2 (108). This work has led to the identification of potential druggable kinases in breast cancer, other than ERBB2, and emphasized the advantage of connecting the genome to the proteome.

### Colorectal Cancer

Zhang et al. analyzed the proteomes of colorectal tumors previously characterized by the TCGA (109). They showed that somatic variants were associated with lower protein abundance compared to germline variants and that mRNA expression did not predict protein abundance between tumors. Proteomics identified five subtypes in the TCGA cohort with two overlapping the transcriptomic subtype: microsatellite instability/CpG island methylation subtype. This demonstrated that proteomic data may enable prioritization of potential driver genes. They also showed that chromosome 20q amplicon was associated with high changes at the mRNA and protein levels, some of which included HNF4α, TOMM34, and SRC (109).

The data obtained from these integrative approaches provide a link from genotype to proteotype to phenotype to better understand the biology at the molecular level that lead to aggressive cancer. Insights gained from the studies mentioned above are evidence that MS-based phosphoproteomics and integration of proteomic and genomic data are advantageous for patient stratification, for identification of personalize therapies, and to understand mechanisms involved in resistance to standard treatment.

## How to Measure Pathway Activity Clinically: Targeted MS

**Figure 4:**
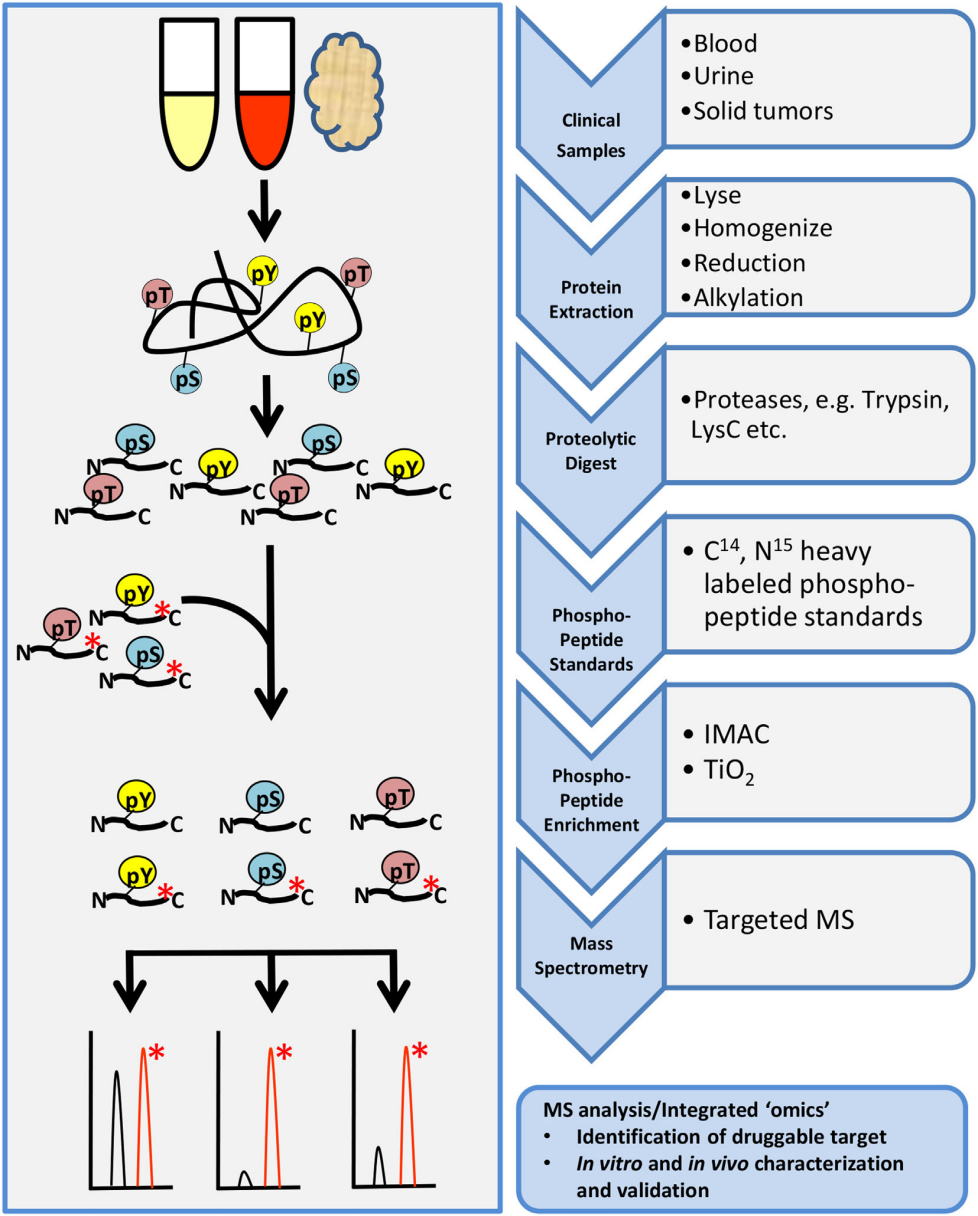
General workflow for targeted phosphoproteomics analysis. Tissue samples may include cultures cell lines, mouse xenografts, or clinical biopsy specimens such as blood, urine, or tumor biopsies. Samples are processed as described in the shotgun workflow up to proteolytic digestion. Custom designed heavy-labeled peptide standards to specific targets of interest are spiked in with the tryptic peptides followed by enrichment and analysis by LC-MS/MS.

Targeted phosphoproteomic technologies are rapidly rising as key tools for the identification and quantification of highly selected phosphopeptides in clinical samples (Figure 4). Some advantages of targeted MS are increased speed, sensitivity, reproducibility, and selectivity of your analyte. Targeted MS technology depends on the use of more expensive heavy-labeled synthetic custom phosphopeptides for assay development and absolute quantification (110–112). These phosphopeptides are designed to limit the focus to a specific subset of targets of interest, without large-scale biomarker screening, although the targets can be highly customizable.

Limited sample amount from biopsies is a major challenge for phosphoproteomic analyses clinically. The optimization of protocols to efficiently sample biopsy amounts coupled with enrichment techniques to reduce sample complexity for reliable and reproducible detection *via* targeted MS are being investigated (42, 113). For example, a phosphoproteomic platform known as EasyPhos was shown to quantify thousands of phosphopeptides from only 1 mg of cells or tissue protein rapidly in a 96-well format (114). In addition, integrating pressure cycling technology-assisted sample preparation and SWATH-MS allowed for consistent sample preparation reproducibility as well as the quantification of thousands of proteins from biopsy level tissues (115, 116). Shao et al. evaluated minimum sample requirements from 50,000 cultured cells and as low as 0.2–0.5 mg of wet tissue weight and reported that these smaller sample sizes achieved high quantitative accuracy that were both reliable and reproducible (115). It is important to note that SWATH-MS was previously applied to the proteome and not the phosphoproteome, so other modifications to the protocol may be necessary. The capability of sample multiplexing using isobaric labeling allows the monitoring of up to 10 samples simultaneously in a single-targeted MS run, significantly reducing cost and run times (117). Overall, with significant improvements and advances in protocol optimization and sample procurement, targeted phosphoproteomic analyses will soon be a feasible and essential tool in the clinical setting for assessment of diagnostic, prognostic, or predictive biomarkers (118).

## Sources of Patient Material for Targeted MS Assays

A number of studies have applied proteomics on various biological systems including tissue, serum (119), urine (120), and cell lines and conditioned media from cultured cells (121). Frozen or formalin-fixed paraffin-embedded samples represent the two major processing protocols for collecting clinical tissues and targeted MS assays are capable to detect proteins or phosphoproteins from either source depending on the assay design. The major challenges with tissue-based approaches are small amounts (biopsies), and they are not conducive for sequential assessment of pathways related to disease progression or drug resistance. Since repeated metastatic tissue biopsies are not feasible, ethical, or safe to patients with mCRPC, assessing liquid biopsies, such as blood or urine, may be an effective substitute for biomarker or pathway evaluation studies over time. Some approaches include isolation of CTCs, cell-free DNA, or exosomes in patient blood or serum.

Cell-free DNA has the capability to detect novel mutations (or loss of a mutation) after treatment, indicative of therapy success or resistance. However, cell-free DNA will generate molecular characterization at the genomic level without any information at the protein level. Several great reviews have been written on cell-free DNA and will not be discussed further (122–124). Isolation of CTCs have also garnered much interest in the PrCa community since the finding that AR splice variants can serve as potential biomarkers of resistance to abiraterone acetate or enzalutamide (14). Currently, CTCs are primarily used for RNA or DNA-based analyses and protocols are being developed to investigate proteins. Indeed, a recent study by Scher et al. identified nuclear ARv7 protein in CTCs of patients with mCRPC as a biomarker for treatment selection (125). Importantly, patients with CTCs consisting of nuclear ARv7 were likely to have a better overall survival on taxane-based chemotherapy, suggesting that the assessment of ARv7 protein is critical for treatment selection. However, assessment of phosphoproteins in CTCs have not been reported and represents a big hurdle is the utilization of this technology to measure pathway activity. Furthermore, isolation of CTCs from blood is technically challenging and the number of CTCs in blood is quite low with counts ranging between 5 and 10 CTCs per 10 ml of blood in patients with low number metastases (126), making this approach unlikely for targeted MS assays in the current form and more suitable for immunofluorescence at the single cell level. However, if we can evaluate signaling networks in CTCs using phosphosite-specific antibodies *via* immunofluorescence, we can gain a better understanding of the heterogeneity of signaling in these cell types as well as identify possible new therapeutic targets for treatment.

An exciting new area in liquid biopsy research that has potential for MS-based assays is the isolation of extracellular vesicles or exosomes. Exosomes are an excellent source for biomarker discovery because the cargo they carry reflects the same genomic, transcriptomic, or proteomic information from parental cancer cells (127, 128). In a study where exosomes were isolated by ultracentrifugation from primary prostate epithelial and PrCa cell line supernatants, MS-based proteomic analysis revealed candidate biomarkers more abundant in PrCa cell lines, including FASN, XPO1, ALIX, and ENO1 (129). In a later MS-based study assessing the proteome of urinary exosomes, differential protein expression was observed between PrCa patients and healthy male controls (130). Some of these proteins included transmembrane protein 256 (TM256), LAMTOR, VATL, ADIRF, and RAB family. Claudin 3 was found in exosomes isolated from the plasma of patients with localized and mCRPC compared to patients with benign prostatic hyperplasia and healthy individuals (131). This demonstrated the benefits and potential clinical application of exosomes for the identification and validation of urine- and blood-based biomarkers in PrCa.

## Conclusion

**Figure 5:**
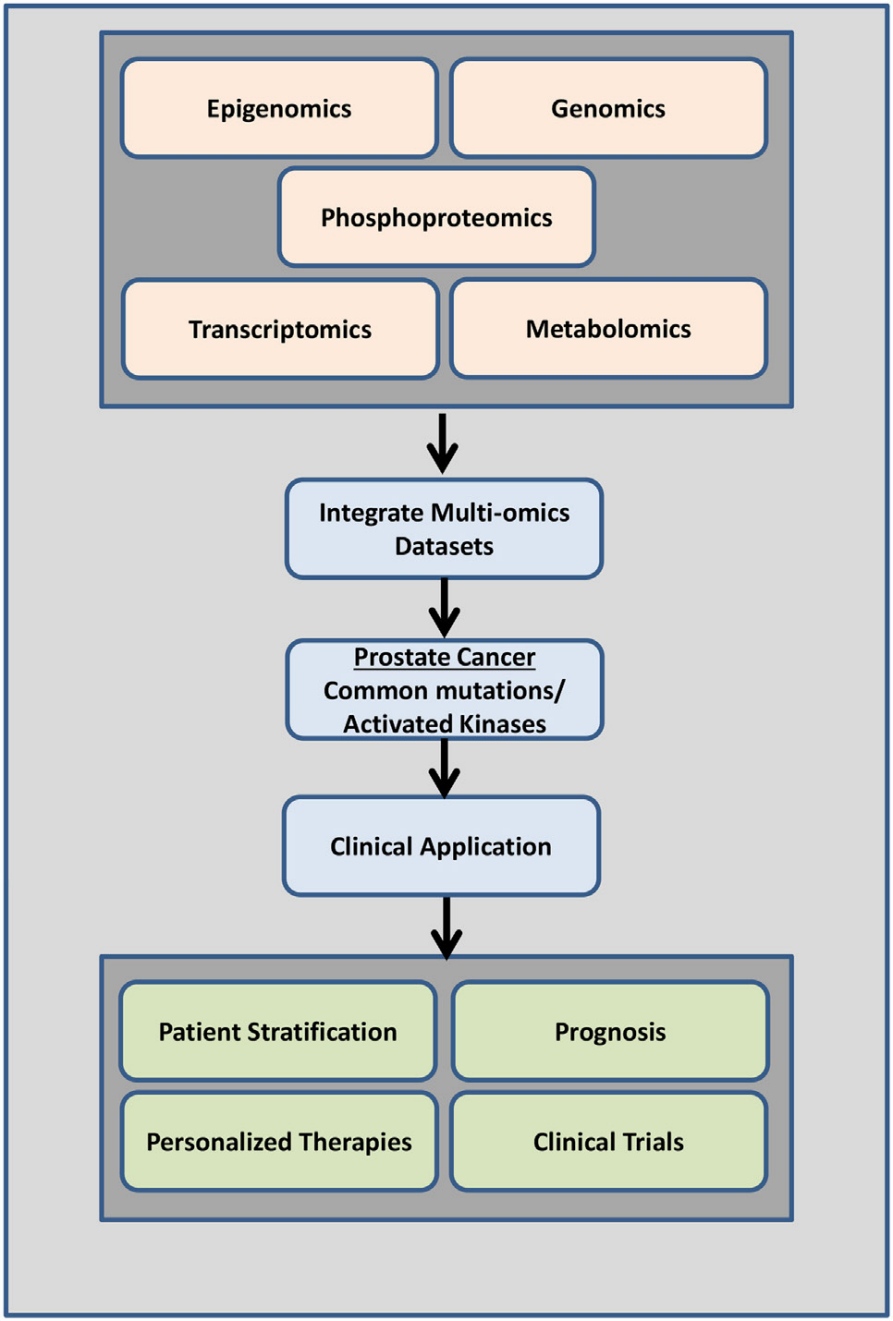
Overview of data integration. Data from a combination of phosphoproteomics, genomics, transcriptomics epigenomics, and metabolomics studies investigating the mutational landscape, phosphoproteomic signature, gene expression changes, and regulation in prostate cancer tumors of individual patients can be used clinically to determine disease drivers (mutations and/or activated kinases and aberrantly regulated signaling pathways) as diagnostic tools, to predict patient outcome, to design personalized therapeutic options, and to aid in better clinical trials design.

The majority of studies have predominantly focused on the genetic signatures of cancer identifying driver mutations that confer drug resistance. Given the low mutation rates in PrCa and mCRPC relative to other cancers strongly supports the need to identify other candidate biomarkers *via* proteomic or phosphoproteomic technologies. Systems biology approaches revealed that genomic and transcriptomic data alone may be missing key players regulating cellular function and disease (132). Integrating other “omics” data sets such as metabolomics, epigenomics, proteomics, and phosphoproteomics with genomic or gene expression data sets will reveal other key pathways, regulators, and complex signaling networks that may be involved in cell growth, proliferation, and cancer progression (Figure 5). This will give us a more comprehensive view into aberrantly regulated signaling pathways and will lead to the identification of key druggable regulators/molecules (Figure 5).

Our understanding of PrCa biology is rapidly increasing, as well as the availability and affordability of high-throughput technologies for comprehensive molecular characterization of PrCa and an individual’s genetic and proteomic makeup. With our expanding knowledge of the key players contributing to PrCa progression and resistance to treatment, the future will provide endless possibilities for rational, personalized therapies. This can be accomplished based on the measured disease drivers (mutations and/or activated kinases and aberrantly regulated signaling pathways) *via* the integration of phosphoproteomic, genomic, and transcriptomic data sets. Continued advancement and development of proteomic and phosphoproteomic technologies will provide exciting new opportunities for molecularly targeted treatment of mCRPC.

## Author Contributions

JR, MS, and JD wrote the commentary. JR and JD prepared and edited the figures.

## Conflict of Interest Statement

The authors declare that the research was conducted in the absence of any commercial or financial relationships that could be construed as a potential conflict of interest.
